# circCCT3 Enhances Invasion and Epithelial-Mesenchymal Transition (EMT) of Non-Small-Cell Lung Cancer (NSCLC) via the miR-107/Wnt/FGF7 Axis

**DOI:** 10.1155/2022/7020774

**Published:** 2022-06-22

**Authors:** Jinyou Li, Rongguo Lu, Kejia Yang, Qi Sun

**Affiliations:** ^1^Department of Thoracic Surgery, Affiliated Hospital of Jiangnan University, Wuxi, China; ^2^Department of Thoracic Surgery, Wuxi People's Hospital, Wuxi, China

## Abstract

**Background:**

CircRNAs play a role in a variety of biological processes, including tumorigenesis. circCCT3 has been shown to regulate cancer initiation and progression. Unfortunately, whether circCCT3 is involved in non-small-cell lung cancer (NSCLC) metastasis remains unclear.

**Methods:**

Our study utilized RT-qPCR to examine gene expression levels. A transwell assay was used to measure invasion ability of cells. Starbase software and TargetScan software were used to predict target genes.

**Results:**

circCCT3 knockdown attenuated invasion and epithelial-mesenchymal transition (EMT) of A549 and Calu-1 cells. miR-107 mimics could rescue circCCT3-induced invasion and EMT. Next, miR-107 mimics and circCCT3 knockdown suppressed Wnt3a and FGF7 expression. An miR-107 inhibitor promoted Wnt3a and FGF7 expressions. Finally, FGF7 greatly restored miR-107-inhibited invasion and EMT of A549 cells.

**Conclusion:**

Here, we reveal a molecular mechanism circCCT3/miR-107/Wnt/FGF7 responsible for NSCLC metastasis.

## 1. Background

Lung cancer includes small-cell lung cancer (SCLC) and non-small-cell lung cancer (NSCLC). NSCLC accounts for about 80% of lung cancer cases [1, 2]. Surgery, chemotherapy, radiotherapy, and targeted therapy are the main treatment methods for NSCLC [3]. Most patients are at the advanced stage with a low 5-year survival rate (about 11%) [4]. Invasion and metastasis are the major causes for NSCLC progression and failure of therapy [5]. Hence, it is essential to illuminate the mechanism underlying NSCLC metastasis.

CircRNAs are defined as single-strand closed RNAs via back splicing of premRNAs [6]. Recently, circRNAs have been reported to modulate tumor progression [7]. circCCT3 is formed from chaperonin containing TCP1 subunit 3 (CCT3) [8]. Although circCCT3 is involved in tumorigenesis and metastasis of colorectal cancer (CRC) and hepatocellular carcinoma (HCC), the potential mechanism by which circCCT3 regulates metastasis of NSCLC remains to be clarified [9, 10].

miRNAs are a class of small noncoding RNAs with 22 nucleotides [11]. They bind to 3′-UTR of mRNAs to downregulate mRNA levels or hamper translation. A number of studies revealed that miRNAs regulated tumor proliferation, invasion, and EMT by downstream genes [12]. miR-107 was recently shown to play a tumor-suppressive role in various cancers, including CRC and NSCLC [13, 14].

Fibroblast growth factors (FGFs) are a family of heparin-binding growth factors [15]. They are implicated in tumor invasion and metastasis [16]. FGF7 is also known as a keratinocyte growth factor or SDGF-3 [15]. FGFR2 is the cognate receptor for FGF7 [17]. Previous studies showed that FGF7 was associated with cervical, gastric, pancreatic, and lung cancer [18–21].

In this study, the purpose is to investigate the role of circCCT3 in NSCLC. Our result will help understand the metastasis of NSCLC and develop potential therapeutic methods.

## 2. Materials and Methods

### 2.1. Cell Culture

Human NSCLC cells (A549 and Calu-1) and human embryonic kidney cell 293T were all purchased from the Cell Bank of the Chinese Academy of Sciences. All cells were cultured using Dulbecco's modified Eagle's medium (DMEM) supplemented with 10% FBS and 1% penicillin/streptomycin. The culture condition is 37°C, 5% CO_2_.

### 2.2. Transfection

miR-107 mimic (50 uM), inhibitor (50 uM), pcDNA3.1-circCCT3, or pcDNA3.1-FGF7 were transiently transfected into A549 and Calu-1 cells using Lipo3000 reagent (Invitrogen). The cells were harvested and used to analyze after 48 h of transfection. For stable cell line generation, sh-circCCT3-1 and sh-circCCT3-2 were used to stably downregulate circCCT3 expression in A549 and Calu-1 cells. shRNAs were subcloned into pLKO.1 and transfected into 293T cells with pVSVG and pPAX2. After 48 h, the lentivirus was harvested and used to infect the NSCLC cells for additional 48 h. Next, 2 ug/ml puromycin was used to select the resistant cells.

  sh-NC: UUGAACCGCAUCCGAAUUUA
  sh-circCCT3-1: GUCAUUUGAGAAAGUUGCCAA
  sh-circCCT3-2: AUUUGAGAAAGUUGCCAAGCA
  NC mimic: AUUCCGCUCAAGCAUUACGG
  miR-107 mimic: AAUCAGGCAUUCAGUCCAUGG
  NC inhibitor: CAUCCGAUUCCAUGGAUUGGA
  miR-107 inhibitor: CAUUGCGAUCUUAGGCUAAGG

### 2.3. Transwell Assay

A549 and Calu-1 cells (1 × 10^4^ cells) were suspended in 150 *µ*L medium without FBS. An upper chamber with 8 *μ*m pore and matrigel-coated membranes was used in this experiment. Then, 150 *µ*L culture media plus 10% FBS were added to the bottom chamber. After 24 h, the invaded cells were stained with 0.005% of crystal violet for 1-2 h at room temperature.

### 2.4. Reverse Transcription-Quantitative PCR (RT-qPCR)

Total RNAs of NSCLC cells were extracted using TRIzol (Invitrogen). Besides, about 1 *µ*g of total RNAs was used to make cDNAs using the PrimeScript RT reagent kit (Takara). Quantitative real-time PCR was performed with the SYBR Green reagent. Relative gene expressions were calculated by the 2^−ΔΔCt^ method.

  circCCT3-Forward: 5′-GCCCATGCCTTGACAGAAAA-3′,
  circCCT3-Reverse: 5′-TCCACCAAAGTACCCGTCTC-3′;
  miR-107-Forward: 5′-TCTTTACAGTGTTGCCTTGTGG-3′,
  miR-107-Reverse: 5′-CCCTGTACAATGCTGCTTGA-3′;
  FGF7-Forward: 5′-TTGTGGCAATCAAAGGGGTG-3′,
  FGF7-Reverse: 5′-CATTTCCCCTCCGTTGTGTG-3′;
  E-cadherin-Forward: 5′-GTCTGTCATGGAAGGTGCT-3′;
  F-cadherin-Reverse: 5′-TACGACGTTAGCCTCGTTC-3′;
  vimentin-Forward: 5′-AGCCGAAAACACCCTGCAAT-3′;
  vimentin-Reverse: 5′-CGTTCAAGGTCAAGACGTGC-3′;
  wnt3a-Forward: 5′-ATTGAATTTGGAGGAATGGT-3′;
  wnt3a-Reverse: 5′-CTTGAAGTACGTGTAACGTG-3′;
 
*β*-actin-Forward: 5′-TGGCATCCACGAAACTACCT-3′
 
*β*-Actin-Reverse: 5′-TCTCCTTCTGCATCCTGTCG-3′.
  U6-Forward: 5′-AGAG CCTGTGGTGTCCG-3′;
  U6-Reverse: 5′-CATCTTCAAAGCACTTCCCT-3′.

### 2.5. Statistical Analysis

Statistical analyses were conducted using Graphpad 6.0 according to data of 3 replicates. Comparisons of two or multiple groups were accomplished by unpaired Student's *t*-test or ANOVA (Tukey's post hoc test). *P* < 0.05 was considered statistically significant.

## 3. Results

### 3.1. circCCT3 Knockdown Attenuated NSCLC Invasion and EMT

**Figure 1:**
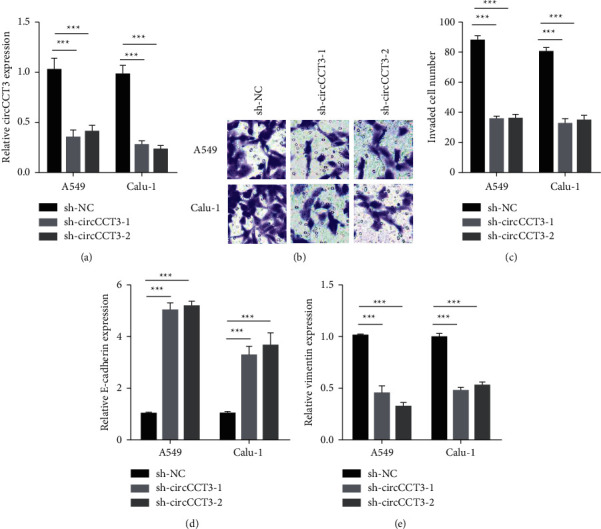
circCCT3 knockdown attenuated NSCLC invasion and EMT. (a) circCCT3 levels were measured by RT-qPCR in A549 and Calu-1 cells stably transfected with sh-NC, sh-circCCT3-1, and sh-circCCT3-2. ^*∗∗∗*^*P* < 0.001^*∗∗∗*^, sh-circCCT3-1 vs sh-NC group, and sh-circCCT3-2 vs sh-NC group. (b, c) Cell invasion was examined in A549 and Calu-1 cells stably transfected with sh-NC, sh-circCCT3-1, and sh-circCCT3-2. ^*∗∗∗*^*P* < 0.001, sh-circCCT3-1 vs sh-NC group, and sh-circCCT3-2 vs sh-NC group. (d, e) E-cadherin and vimentin levels were measured by RT-qPCR in A549 and Calu-1 cells stably transfected with sh-NC, sh-circCCT3-1, and sh-circCCT3-2. ^*∗∗∗*^*P* < 0.001, sh-circCCT3-1 vs sh-NC group, and sh-circCCT3-2 vs sh-NC group.

To investigate the role of circCCT3 in metastasis of NSCLC cells, we designed sh-RNAs against circCCT3 to generate stable cell lines. circCCT3 levels were markedly decreased in A549 or Calu-1 cells stably expressing sh-circCCT3-1 or sh-circCCT3-2 (Figure 1(a)). The transwell assay data demonstrated that sh-circCCT3-1 and sh-circCCT3-2 cells exhibited impaired invasive ability of cells compared to sh-NC cells (Figures 1(b) and 1(c)). More interestingly, sh-circCCT3-1 and sh-circCCT3-2 caused the increased E-cadherin level and the decreased vimentin level in both A549 and Calu-1 cells (Figures 1(d) and 1(e)). Taken together, our results indicated that circCCT3 knockdown attenuated invasion and EMT of NSCLC cells.

### 3.2. mMiR-107 Mimic Rescued circCCT3-Induced Invasion and EMT of NSCLC Cell

**Figure 2:**
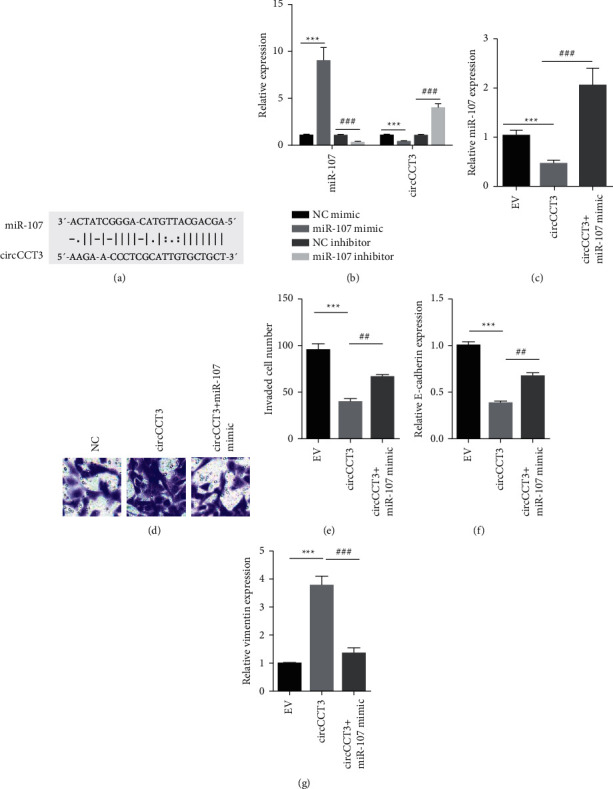
The miR-107 mimic rescued circCCT3-induced invasion and EMT of NSCLC cell. (a) StarBase 2.0 predicted miR-107 as the potential target for circCCT3. (b) RT-qPCR showed miR-107 and circCCT3 levels in A549 cells transiently transfected with the NC mimic, miR-107 mimic, NC inhibitor, and miR-107 inhibitor. ^*∗∗∗*^*P* < 0.001, miR-107 mimic vs NC mimic group; ^###^*P* < 0.001, circTTC3 + miR-107 mimic vs circTTC3 group. (c) RT-qPCR showed miR-107 levels in A549 cells transiently transfected with pcDNA3.1 (EV) and pcDNA3.1-circCCT3 (circCCT3) and pcDNA3.1-circCCT3 plus miR-107 mimic. ^*∗∗∗*^*P* < 0.001, circTT3C vs EV group; ^###^*P* < 0.001, circTTC3 + miR-107 mimic vs circTTC3 group. (d, e) Cell invasion was examined in A549 cells transiently transfected with pcDNA3.1 (EV) and pcDNA3.1-circCCT3 (circCCT3) and pcDNA3.1-circCCT3 plus miR-107 mimic. ^*∗∗∗*^*P* < 0.001, miR-107 mimic vs NC mimic group; ^##^*P* < 0.01, circTTC3 + miR-107 mimic vs circTTC3 group. (f, g) E-cadherin and vimentin levels were measured by RT-qPCR in A549 cells transiently transfected with pcDNA3.1 (EV) and pcDNA3.1-circCCT3 (circCCT3) and pcDNA3.1-circCCT3 plus miR-107 mimic. ^*∗∗∗*^*P* < 0.001, miR-107 mimic vs NC mimic group; ^##^*P* < 0.01, ^###^*P* < 0.001, circTTC3 + miR-107 mimic vs circTTC3 group.

To identify the potential target of circCCT3, Starbase 2.0 was used to predict miR-107 that was the most likely target (Figure 2(a)). miR-107 mimic transfection led to lower circCCT3 expression, while the miR-107 inhibitor resulted in higher circCCT3 expression in A549 cells (Figure 2(b)). Moreover, circCCT3 reduced the miR-107 level in A549 cells, which was partially elevated in cells transfected with circCCT3 plus miR-107 mimic compared to the circCCT3 group (Figure 2(c)). The transwell assay showed that circCCT3 overexpression stimulated invasive ability of A549 cells compared to that of the EV group. The miR-107 mimic attenuated circCCT3-induced cell invasion (Figures 2(d) and 2(e)). Besides, circCCT3 overexpression caused the decreased E-cadherin level and the increased vimentin level in A549 cells, which was reverted by the miR-107 mimic (Figures 2(f) and 2(g)). Together, our results indicated that miR-107 served as the target of circCCT3 in NSCLC cells.

### 3.3. circCCT3 and miR-107 Regulated the Wnt Signaling Pathway and FGF7

**Figure 3:**
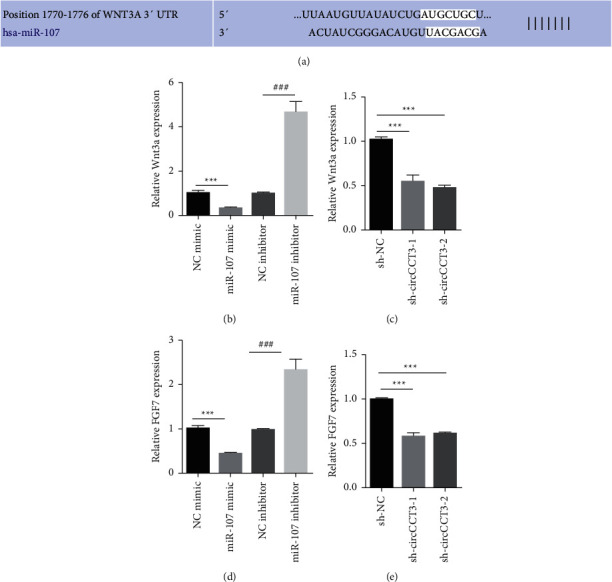
circCCT3 and miR-107 regulated the Wnt signaling pathway and FGF7. (a) TargetScan predicted Wnt3a as the potential target for miR-107. (b, d) RT-qPCR showed Wnt3a and FGF7 levels in A549 cells transiently transfected with the NC mimic, miR-107 mimic, NC inhibitor, and miR-107 inhibitor. ^*∗∗∗*^*P* < 0.001, miR-107 mimic vs NC mimic group; ^###^*P* < 0.001, miR-107 inhibitor vs NC inhibitor group. (c, e) Wnt3a and FGF7 levels were measured by RT-qPCR in A549 cells stably transfected with sh-NC, sh-circCCT3-1, and sh-circCCT3-2. ^*∗∗∗*^*P* < 0.001, sh-circCCT3-1 vs sh-NC group, and sh-circCCT3-2 vs sh-NC group.

Next, we sought to figure out the downstream effectors for circCCT3/miR-107. TargetScan software was utilized to identify Wnt3a that was the possible target of miR-107 (Figure 3(a)). miR-107 mimic transfection led to downregulation of Wnt3a, while the miR-107 inhibitor resulted in upregulation of Wnt3a in A549 cells (Figure 3(b)). circCCT3 knockdown significantly reduced Wnt3a expression levels (Figure 3(c)). As FGF7 was one of the target genes of the Wnt signaling pathway, we hypothesized that FGF7 was important for circCCT3/miR-107-mediated invasion and EMT of NSCLC cells. The RT-qPCR data suggested that the miR-107 mimic downregulated FGF7 expression, while the miR-107 inhibitor upregulated FGF7 expression in A549 cells (Figure 3(d)). circCCT3 knockdown greatly reduced FGF7 expression levels (Figure 3(e)). In summary, our data suggested that circCCT3 and miR-107 regulated the Wnt pathway and FGF7.

### 3.4. FGF7 Acted as a Key Effector for miR-107 in NSCLC Cell

**Figure 4:**
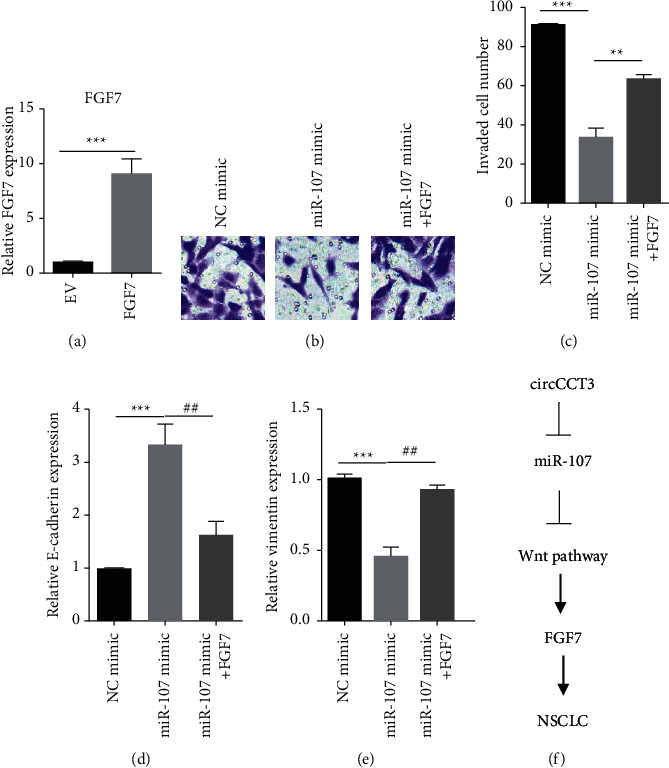
FGF7 acted as a key effector for miR-107 in NSCLC cell. (a) FGF7 levels were examined by RT-qPCR in A549 cells transiently transfected with pcDNA3.1 (EV) and pcDNA3.1-FGF7 (FGF7). ^*∗∗∗*^*P* < 0.001, FGF7 vs EV group. (b, c) Cell invasion was examined in A549 cells transiently transfected with the NC mimic, miR-107 mimic, and miR-107 mimic plus pcDNA3.1-FGF7. ^*∗∗∗*^*P* < 0.001, miR-107 mimic vs NC mimic group; ^###^*P* < 0.001, miR-107 inhibitor vs NC inhibitor group. (d, e) E-cadherin and vimentin levels were measured by RT-qPCR in A549 cells transiently transfected with the NC mimic, miR-107 mimic, and miR-107 mimic plus pcDNA3.1-FGF7. ^*∗∗∗*^*P* < 0.001, miR-107 mimic vs NC mimic group; ^##^*P* < 0.01, miR-107 + FGF7 vs miR-107 mimic group. (f) The model showing a molecular mechanism underlying circCCT3-mediated metastasis of NSCLC.

In order to determine whether FGF7 could play a role in circCCT3/miR-107-mediated invasion and EMT, FGF7 was overexpressed in A549 cells (Figure 4(a)). Expectedly, FGF7 promoted miR-107-inhibited invasion of A549 cells (Figures 4(b) and 4(c)). In addition, the RT-qPCR data revealed that the miR-107 mimic increased the level of E-cadherin and decreased the level of vimentin, whereas FGF7 overexpression partially reverted these changes (Figures 4(d) and 4(e)). Collectively, FGF7 acted as a key effector for circCCT3/miR-107-mediated invasion and EMT of NSCLC cells.

In many studies, circRNAs modulated metastasis of NSCLC; however, the biological role of circCCT3 was unknown. Our research revealed that circCCT3 interacted with miR-107. Besides, the Wnt signaling and FGF7 were the key effectors for circCCT3/miR-107 (Figure 4(f)).

## 4. Discussion

As the biomarker and treatment option of NSCLC were scarce, the targeted therapy might be promising. circRNAs were shown to regulate NSCLC progression. For instance, circFGFR1 stimulated NSCLC cell migration, invasion, and immune evasion [22]. circPTPRA inhibited EMT and metastasis of NSCLC [23]. In addition, circ_0000376 enhanced metastasis and drug resistance of NSCLC cells [24]. circCCT3 was upregulated in different types of cancers and associated with prognosis. In previous reports, circCCT3 sponged miR-613 via acting as competing endogenous RNA (ceRNA) [9]. In addition, we used bioinformatic and biochemical approaches to identify circCCT3 as ceRNA of miR-107 in this paper. Moreover, miR-107 negatively regulated the circCCT3 expression level.

miRNAs play a role in NSCLC metastasis and EMT. MiR-107 suppressed HCC proliferation and metastasis through regulation of the Wnt signaling pathway [25]. Zhang et al. also showed this regulatory event in preadipocytes [26]. Especially, miR-107 could serve as an important downstream effector for lncRNAs or circRNAs in NSCLC [3]. As for key targets of miR-107, BDNF, CDK6, FGFRL1, and STK33 were confirmed in vitro and in vivo [13, 27–29]. In our study, we showed Wnt3a was the crucial target of miR-107, which enriched our understanding of how miR-107 inhibited invasion and EMT of NSCLC cells.

FGF7 generally functions as an oncogene in tumors. Prior research showed FGF7 was a target of Wnt signaling [30]. Our data indicated FGF7 could play a tumor-suppressive role in miR-107. This finding was in agreement with the published results [13, 14]. In the future, other targets of miR-107 need to be discovered and the function should be investigated.

## 5. Conclusion

In summary, we figure out the underlying mechanism of circCCT3 for invasion and EMT of NSCLC. These data help gain our insights into NSCLC progression and develop the targeted therapy.

## Data Availability

The underlying data supporting the results of this study can be obtained from the corresponding author.
